# Supplementary material for: SKIP‐HOPS recruits TBC1D15 for a Rab7‐to‐Arl8b identity switch to control late endosome transport
Source: EMBO J. 2020 Feb 21;39(6):e102301. doi: 10.15252/embj.2019102301 (PMC7073467; doi:10.15252/embj.2019102301)
Supplement: Supplementary file 1 — Expanded View Figures PDF [file EMBJ-39-e102301-s001.pdf]

Expanded View Figures

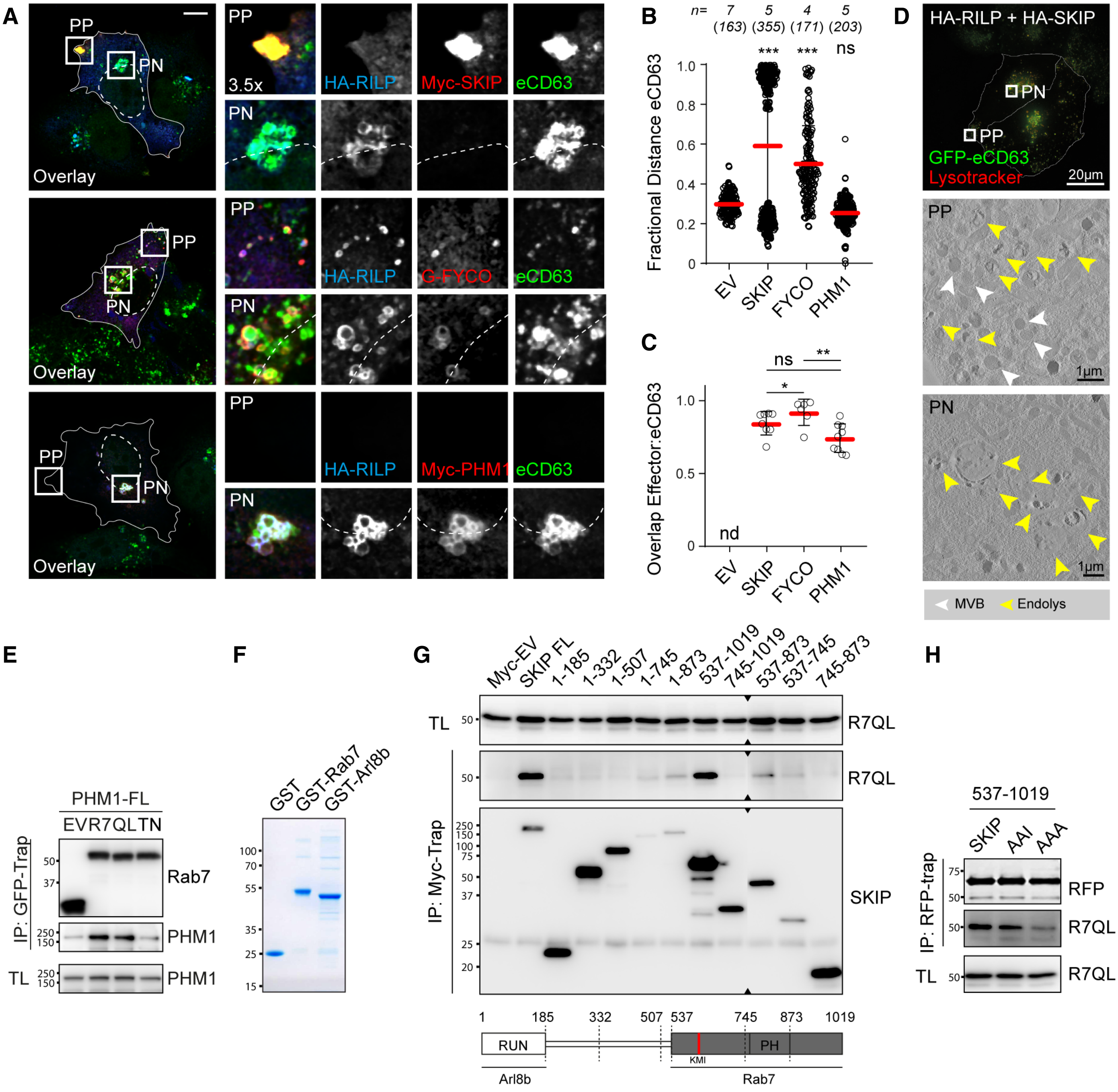

Figure EV1.

 © 2020 The Authors

Figure EV1. Late compartment segregation by Arl8b and Rab7 effectors SKIP and RILP (related to Figs 2–4).

A Representative confocal images of fixed HeLa cells ectopically expressing HA-RILP (*blue*) in combination with Myc-SKIP, GFP-FYCO or Myc-PLEKHM1 (*red*), immunolabelled against endogenous CD63 (*green*) and the indicated epitope tags. Zoom insets (3.5×) highlight select peripheral (PP) and perinuclear (PN) cell regions, scale bar: 10 μm.

B Plots of CD63 pixel distribution as a function of various effector perturbations shown in (A) expressed as fractional distance along a straight line from the centre of the nucleus (0) to the cell membrane (1.0), number of (pixels) plotted given above each scatter, $n \geq 4$ cells per condition analysed from 2 independent experiments. Significance: one-way ANOVA (relative to EV), ***$P < 0.001$, ns: not significant.

C Colocalization (Mander's overlap) of the indicated effectors with CD63, $n \geq 6$ images ($2 \geq$ cells per image) per condition analysed from 2 independent experiments. Significance: 2-tailed Student's *t*-test, *$P < 0.05$, **$P < 0.01$, ns: not significant, nd: not determined.

D *Upper panel:* wide-field image of fixed HeLa cells harbouring endogenous CD63 tagged with GFP, co-transfected with HA-RILP and HA-SKIP and labelled with SiR-lysosome. Selected tomogram slices for peripheral (PP, *middle panel*) and perinuclear (PN, *bottom panel*) cell regions are shown (see also Movies EV1 and EV2). Arrowheads designate distinct endosomal subtypes: MVBs (*white)* and endolysosomes (*yellow*), scale bars as indicated.

E Co-immunoprecipitations (Co-IP) of PLEKHM1-FLAG with GFP-Rab7 (R7) versus its mutants Q67L (QL) and T22N (TN) from HEK293T cells using GFP-trap beads. Representative immunoblots against GFP and Flag are shown; EV: empty vector, TL: total lysate.

F InstantBlue staining of purified GST, GST-Rab7 and GST-Arl8b proteins.

G Myc-SKIP truncation analysis for interactions with GFP-Rab7 Q67L by Co-IP from HEK293T cells using Myc-trap beads. Representative immunoblots against Myc and GFP are shown, along with a schematic representation of SKIP domain organization. Regions of SKIP capable of interacting with Arl8b versus Rab7 are demarcated with solid black lines.

H Co-IP of C-terminal RFP-SKIP fragment (aa 537–1,019) versus its KMI motif mutants AAI and AAA with constitutively active GFP-Rab7 Q67L from HEK293T cells using RFP-Trap beads. Representative immunoblots against RFP and GFP are shown.

Data information: cell and nuclear boundaries are demarcated with solid and dashed lines, respectively. Graphs report the mean (red line) of sample values (open circles), error bars reflect ± SD.

Source data are available online for this figure.

Figure EV2. TBC1D15 is a GAP for Rab7 affecting endosomal organization and HLA-DR trafficking (related to Figs 6 and 7).

A–C Consequences of TBC1D15 depletion for the organization of late compartments. (A) Effectiveness of TBC1D15 (pool and single duplexes #1–#4) and TBC1D17 (pool) targeting siRNA oligos as assayed by immunoblot of total lysates (TL) following immunoprecipitation (IP) against TBC1D15. (B) Plots of CD63 pixel distribution as a function of TBC1D15 depletion (pool and single duplexes #1–#4) expressed as fractional distance along a straight line from the centre of the nucleus (0) to the cell membrane (1.0), number of (pixels) plotted given above each scatter, $n \geq 7$ cells analysed per condition from 2 independent experiments. Significance: one-way ANOVA (relative to siC), **$P < 0.01$, ***$P < 0.001$. (C) Colocalization (Mander's overlap) of endogenous Rab7 (top graph) versus Arl8b-GFP (bottom graph) with Myc-SKIP in response to TBC1D15 depletion, $n \geq 4$ images, $2 \geq$ cells per image, analysed from 2 independent experiments. Significance: one-way ANOVA (relative to siC), **$P < 0.01$, ***$P < 0.001$, ns: not significant.

D Effects of depleting different Rab7 GAPs on late compartment segregation. Representative confocal images of fixed HeLa cells transfected with either control siRNA (siC) or oligo pools targeting TBC1D2, TBC1D5, TBC1D15 or TBC1D17 and ectopically expressing HA-RILP (*blue*) and Myc-SKIP (*green*), immunolabelled against endogenous Rab7 (eRab7, *red*) and relevant epitope tags.

E *In vitro* GAP assay showing the effect on γ^{32}P-GTP hydrolysis with increasing concentration of purified TBC domain of TBC1D15 on γ^{32}P-GTP loaded Rab7, Rab5, Ran and Rab9 GTPases. Plotted are hydrolysis rates relative to no TBC1D15 added (1.0).

F Effects of TBC1D15 depletion on trafficking of Rab7-dependent cargo MHC-II to the SKIP-positive compartment. Representative images of fixed MelJuSo cells transfected with either control siRNA (siC) or a pool of oligos targeting TBC1D15 (siTBC1D15) and ectopically expressing Myc-SKIP (*red*) and HA-RILP (*blue*), immunostained against MHC-II (*green*) and relevant epitope tags.

Data information: cell and nuclear boundaries are demarcated with solid and dashed lines, respectively, and zoom insets (3.5×) highlight select peripheral (PP) and perinuclear (PN) cell regions, all scale bars: 10 μm. Graphs report mean (red line) of sample values (open circles), and error bars reflect ± SD.

Source data are available online for this figure.

© 2020 The Authors

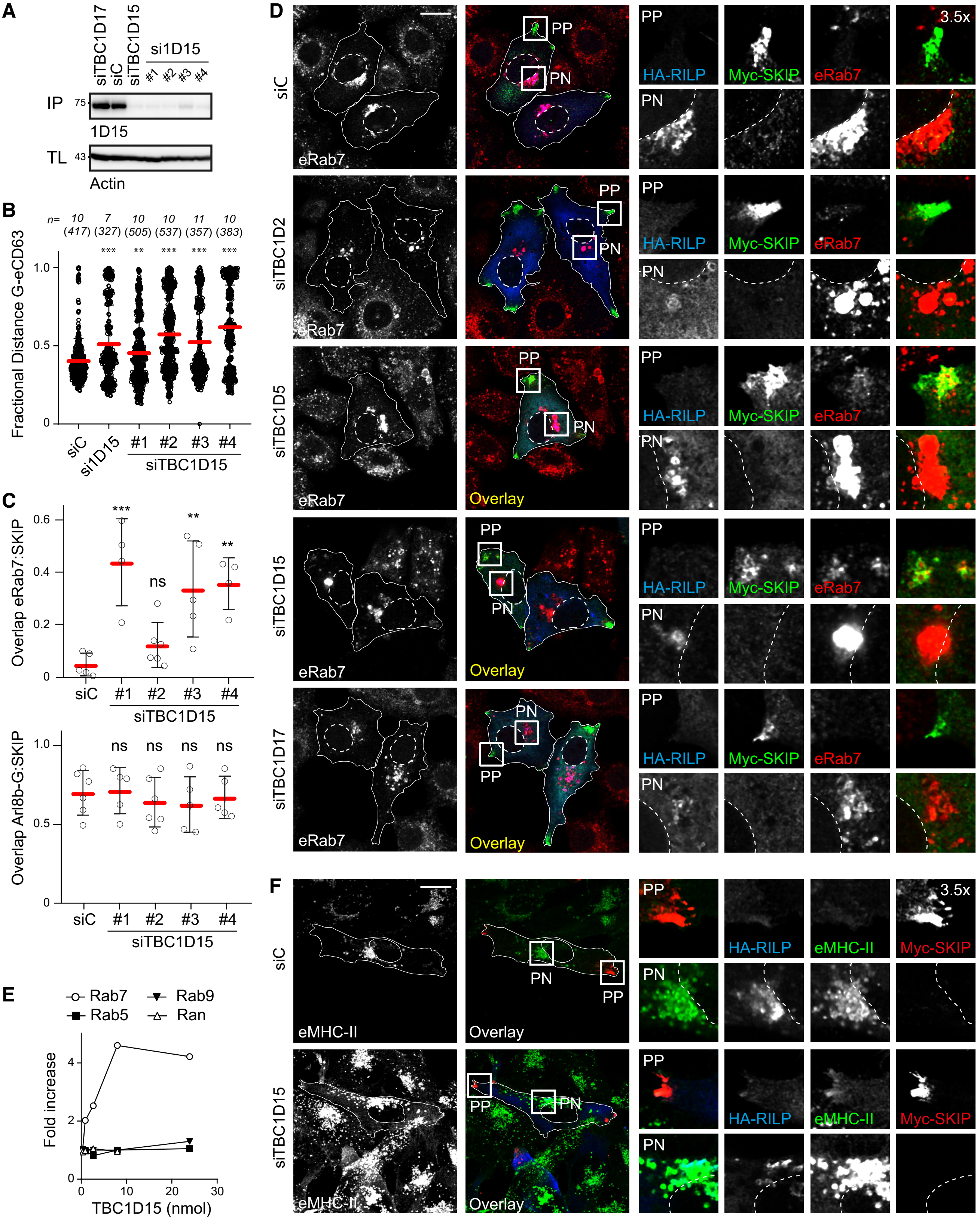

Figure EV2.

© 2020 The Authors

Figure EV3. SKIP recruits non-mitochondria-bound endogenous TBC1D15 (related to Fig 8).

A Validation of endogenously GFP-tagged TBC1D15 in HeLa cells. Representative confocal images of eTBC1D15 (*green*) HeLa cells transfected with either control siRNA (siC) or a pool of oligos targeting TBC1D15 (siTBC1D15), fixed and immunostained against CD63 (*magenta*).

B Fluorescence recovery after photobleaching (FRAP) of endogenous GFP-tagged TBC1D15 in HeLa cells. *Left panel*: per cent of GFP signal recovery over time, n = 3 cells analysed from a representative experiment, error bars reflect ± SD. *Right panels*: representative images taken at the indicated time points, and zoom insets (2.5×) highlight the bleach region.

C Representative confocal images of either control (siC) HeLa cells or those depleted of FIS1 using a pool of siRNA oligos (siFIS1) and ectopically expressing HA-RILP (*blue*) and Myc-SKIP (*green*), fixed and immunolabelled for endogenous Rab7 (*red*) and relevant epitope tags.

D, E Representative confocal images of fixed HeLa cells expressing endogenous GFP-tagged GFP-TBC1D15 (e1D15, *green*) in the presence or absence of HA-SKIP (*blue*), immunostained against HA and (D) Tom20 (*red*) or (E) labelled using Mitotracker (*red*).

Data information: cell and nuclear boundaries are demarcated with solid and dashed lines, respectively, and zoom insets (3.5×) highlight peripheral (PP) and perinuclear (PN) cell regions. All scale bars: 10 μm.

© 2020 The Authors

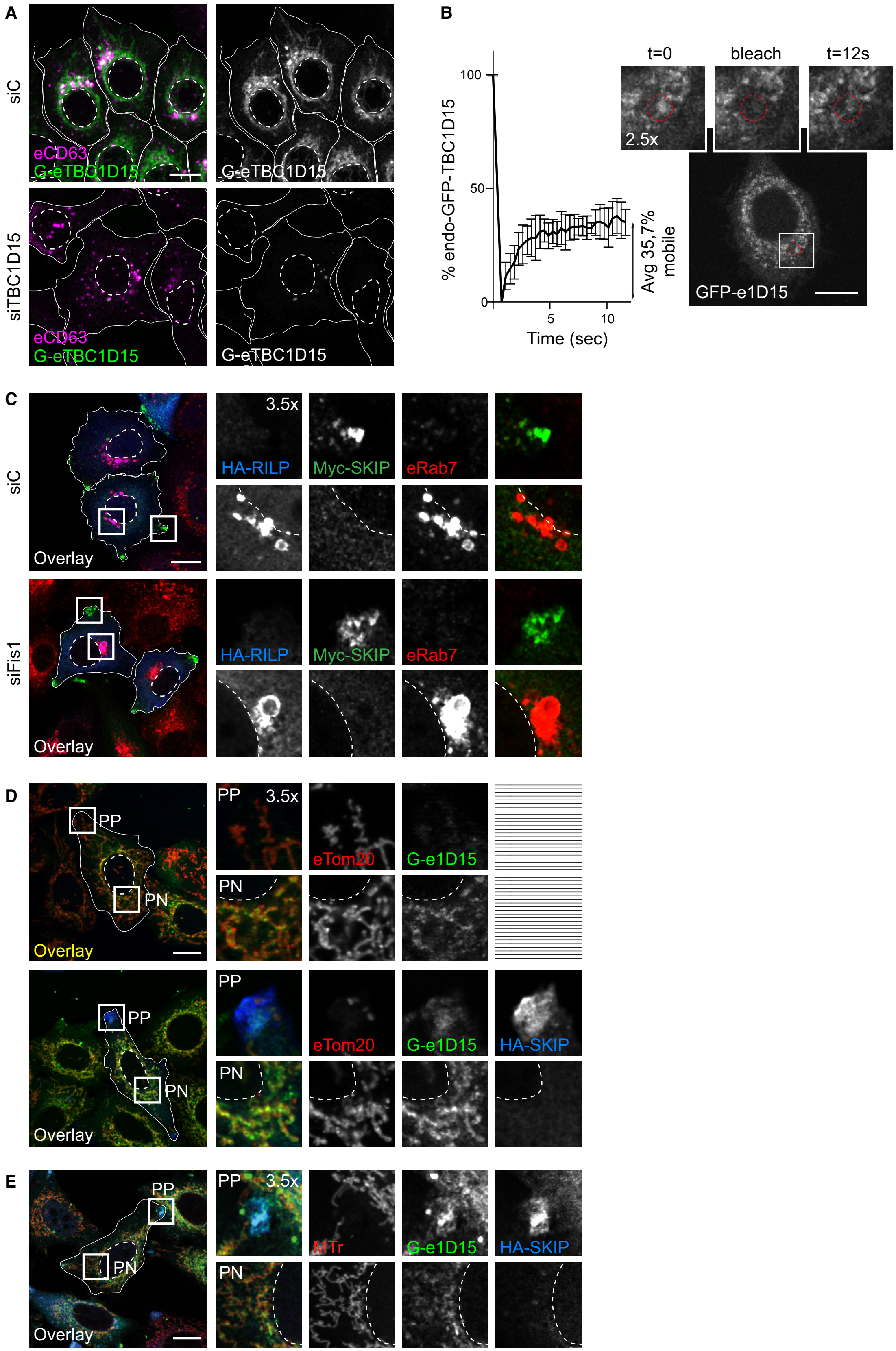

Figure EV3.

 © 2020 The Authors

Figure EV4. GAP TBC1D15 interacts with the HOPS complex to inactivate and remove Rab7 from SKIP-selected membranes (related to Fig 9).

A, B Analysis of HOPS/SKIP interactions by Co-IP of Myc-SKIP truncations with (A) RFP-VPS39 or (B) mCherry-VPS41 from HEK293T cells using Myc-trap beads. Representative immunoblots against RFP/mCherry and Myc are shown.

C Effects of Rab7 and/or Arl8b interactions on the SKIP compartment. Representative confocal images of fixed HeLa cells ectopically expressing Myc-SKIP (*red*), KMI motif mutant (AAA) or RUN domain truncation mutant (ΔRUN) together with mCherry-VPS41 (*green*), immunolabelled for endogenous VPS11 (eVPS11, *blue*). Zoom insets (3.5×) highlight select peripheral (PP) cell regions.

D Effect of HOPS complex member overexpression on recruitment of eTBC1D15 to SKIP. Representative confocal images of fixed HeLa cells harbouring endogenous GFP-TBC1D15 (G-e1D15, *green*) and transfected with HA-SKIP (*red*) and either mCherry-VPS11, VPS16 or VPS41 (*white*), immunostained against endogenous VPS18 (eVPS18, *blue*) and HA. Zoom insets (2.4×) highlight select peripheral (PP) and perinuclear (PN) cell regions.

Data information: cell and nuclear boundaries are demarcated with solid and dashed lines, respectively, all scale bars: 10 μm.

Source data are available online for this figure.

© 2020 The Authors

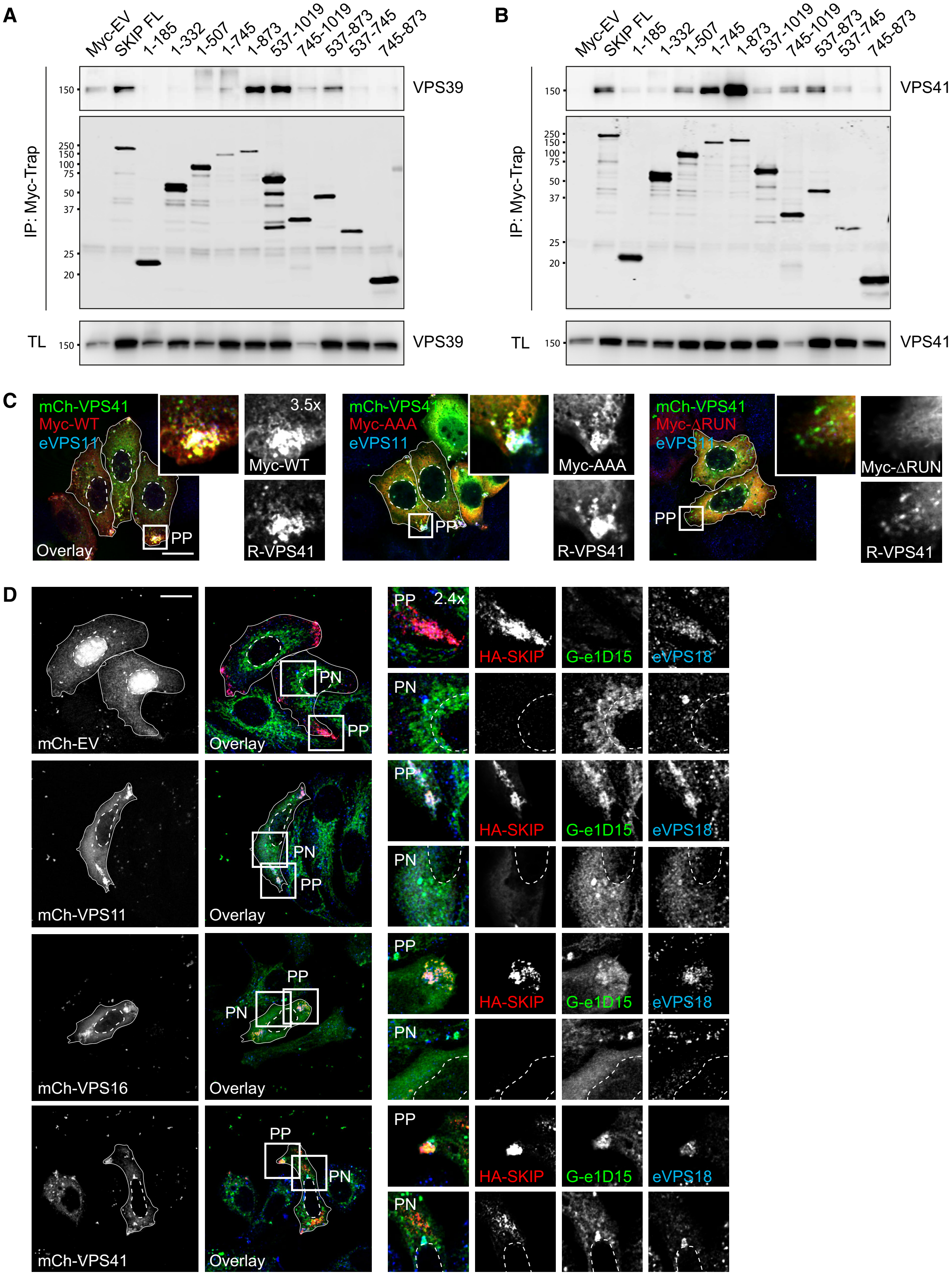

Figure EV4.

 © 2020 The Authors

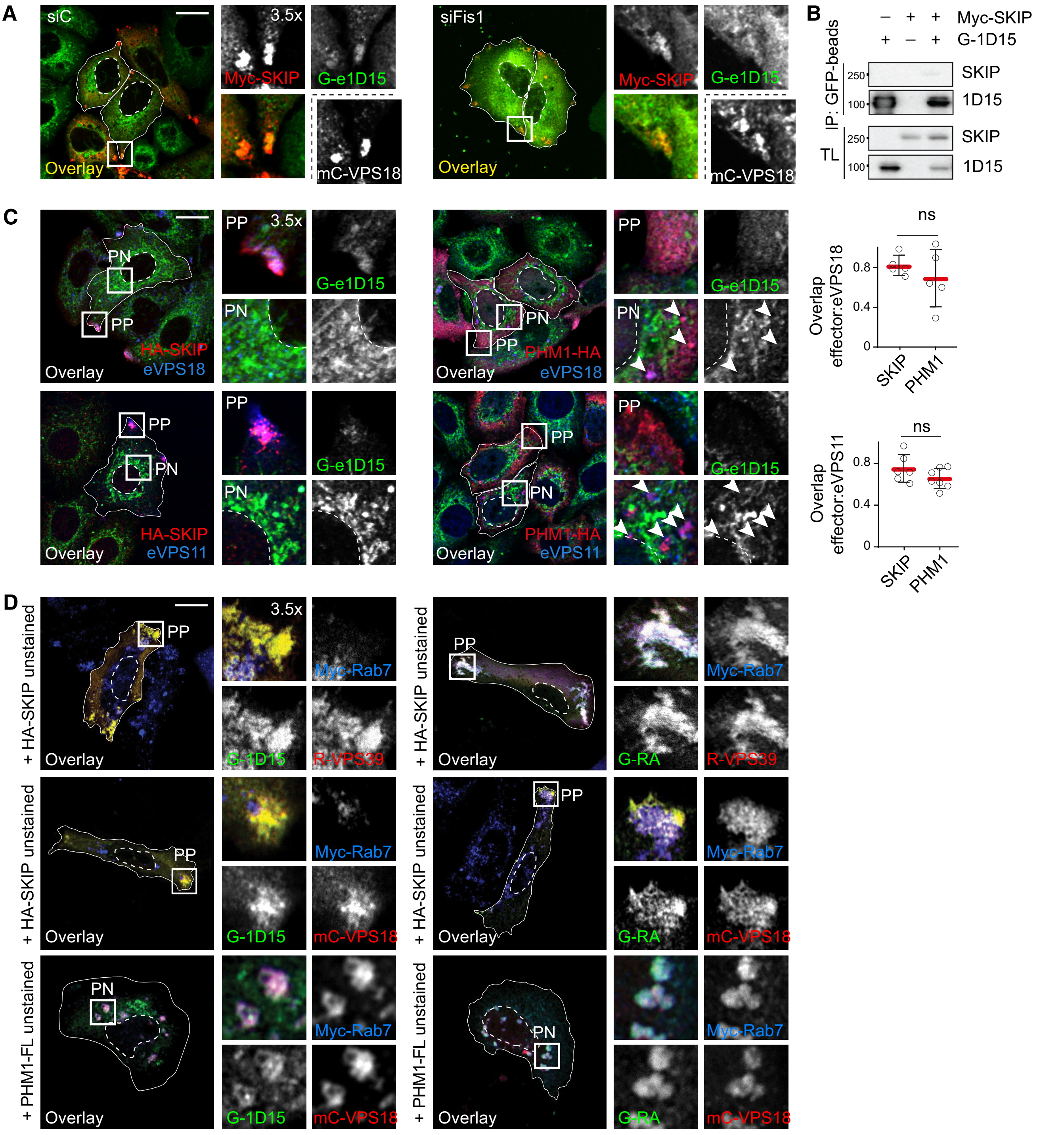
A
siC
3.5x
Myc-SKIP
G-e1D15
Overlay
mC-VPS18
siFis1
B
Myc-SKIP
G-1D15
IP: GFP-beads
TL
SKIP
1D15
250
100
C
PN
PP
HA-SKIP
eVPS18
eVPS11
PHM1-HA
ns
Overlap effector:eVPS18
Overlap effector:eVPS11
SKIP
PHM1
D
+ HA-SKIP unstained
+ PHM1-FL unstained
Myc-Rab7
G-1D15
R-VPS39
mC-VPS18
G-RA

Figure EV5.

Figure EV5. SKIP and PLEKHM1 both interact with the HOPS complex, but only SKIP recruits the Rab7 GAP TBC1D15 (related to Figs 9 and 10).

- A Effect of FIS1 depletion on recruitment of TBC1D15 to the SKIP/HOPS complex. Representative confocal images of either control (siC) HeLa cells harbouring endogenous TBC1D15 tagged with GFP (G-e1D15, *green*) or those depleted of FIS1 using a pool of siRNA oligos, expressing Myc-SKIP (*red*) and mCherry-VPS18, fixed and immunolabelled for Myc.
- B Co-immunoprecipitations (Co-IP) of Myc-SKIP with GFP-TBC1D15 (1D15) from HEK293T cells using GFP-trap beads. Representative immunoblots against Myc and GFP are shown, TL: total lysate.
- C Effect of HOPS complex member overexpression on recruitment of eTBC1D15 to different Rab7 effectors. Representative confocal images of fixed HeLa cells harbouring endogenous GFP-TBC1D15 (G-e1D15, *green*), transfected with HA-SKIP or PLEKHM1-HA (*red*), immunostained against endogenous (e) VPS18 (*blue, upper panels*) or VPS11 (*blue, bottom panels*) and HA. Arrowheads point at PLEKHM1/HOPS-positive, TBC1D15-negative structures. Graphs report fraction overlap (Mander's coefficient) between the indicated Rab7 effectors and endogenous VPS18 or VPS11. Red line: mean of sample values (open circles), $n \geq 5$ images, $2 \geq$ cells per image, analysed from 2 independent experiments, error bars reflect $\pm$ SD. Significance: two-tailed Student's *t*-test, ns: not significant.
- D Representative confocal images of fixed HeLa cells ectopically expressing HA-SKIP (upper and middle panels, *unstained*) or PLEKHM1-FLAG (bottom panels, *unstained*) together with RFP-VPS39 (upper panels, *red*) or mCherry-VPS18 (middle and bottom panels, *red*), Myc-Rab7 (*blue*), and either GFP-TBC1D15 wild type (WT) or its inactive point mutant R417A (RA) (*green*), immunostained against Myc.

Data information: cell and nuclear boundaries are demarcated with solid and dashed lines, respectively, and zoom insets (3.5×) highlight select peripheral (PP) and perinuclear (PN) cell regions. All scale bars: 10 μm.

Source data are available online for this figure.

© 2020 The Authors
