# Supplementary material for: SKIP‐HOPS recruits TBC1D15 for a Rab7‐to‐Arl8b identity switch to control late endosome transport
Source: EMBO J. 2020 Feb 21;39(6):e102301. doi: 10.15252/embj.2019102301 (PMC7073467; doi:10.15252/embj.2019102301)
Supplement: Supplementary file 3 — Movie EV2 [file EMBJ-39-e102301-s003.zip › Movie_Legend_EV2.docx]

**Movie EV2.** **Arl8b-positive endolysosome dynamics in the presence of Rab7 Q67L (*related to Figure 1*).**

Time-lapse (50 s, 0.5 s / frame) of HeLa cells co-expressing Arl8b-GFP (*green*) and mCherry-Rab7 Q67L (*see also Fig 1C-F*).
