# Supplementary material for: SKIP‐HOPS recruits TBC1D15 for a Rab7‐to‐Arl8b identity switch to control late endosome transport
Source: EMBO J. 2020 Feb 21;39(6):e102301. doi: 10.15252/embj.2019102301 (PMC7073467; doi:10.15252/embj.2019102301)
Supplement: Supplementary file 6 — Movie EV5 [file EMBJ-39-e102301-s006.zip › Movie_EV5_Legend.docx]

**Movie EV5.** **Tomogram of the perinuclear region (*related to Figures 3 and EV1*).**

Tomogram depicting endolysosome morphology in the perinuclear region of HeLa cells expressing endogenous GFP-CD63 and exogenous HA-RILP and HA-SKIP (s*ee also Fig EV1D*).
