# Supplementary material for: SKIP‐HOPS recruits TBC1D15 for a Rab7‐to‐Arl8b identity switch to control late endosome transport
Source: EMBO J. 2020 Feb 21;39(6):e102301. doi: 10.15252/embj.2019102301 (PMC7073467; doi:10.15252/embj.2019102301)
Supplement: Supplementary file 9 — Movie EV8 [file EMBJ-39-e102301-s009.zip › Movie_Legend_EV8.docx]

**Movie EV8.** **Recruitment of SKIP to Rab7-positive compartments: example 2 (*related to Figure 4*).**

Time-lapse (23 min, 20 s / frame) of HeLa cells co-expressing GFP-ER-SKIP (*green*) and mCherry-Rab7 (*magenta*) together with HA-RILP (not visualized) expressed at low levels (cells transfected at 1:5 RILP:SKIP ratio) collected following Tamoxifen addition. (*See also Fig 4K*)
