# Supplementary material for: SKIP‐HOPS recruits TBC1D15 for a Rab7‐to‐Arl8b identity switch to control late endosome transport
Source: EMBO J. 2020 Feb 21;39(6):e102301. doi: 10.15252/embj.2019102301 (PMC7073467; doi:10.15252/embj.2019102301)
Supplement: Supplementary file 10 — Movie EV9 [file EMBJ-39-e102301-s010.zip › Movie_Legend_EV9.docx]

**Movie EV9.** **Endolysosome dynamics in control HeLa cells (*related to Figure 6*).**

Time-lapse (8 min, 5 s / frame) of HeLa cells transfected with control siRNA (siC) visualizing late compartment dynamics marked by endogenous GFP-CD63 (*green*) and SiR-Lysosome (*magenta*). (*See also Fig 6G and H*)
