# Supplementary material for: SKIP‐HOPS recruits TBC1D15 for a Rab7‐to‐Arl8b identity switch to control late endosome transport
Source: EMBO J. 2020 Feb 21;39(6):e102301. doi: 10.15252/embj.2019102301 (PMC7073467; doi:10.15252/embj.2019102301)
Supplement: Supplementary file 11 — Movie EV10 [file EMBJ-39-e102301-s011.zip › Movie_Legend_EV10.docx]

**Movie EV10. Endolysosome dynamics in HeLa cells depleted of TBC1D15 (*related to Figure 6*).**

Time-lapse (8 min, 5 s / frame) of HeLa cells transfected with siRNA oligo pool targeting TBC1D15 (siTBC1D15) visualizing late compartment dynamics marked by endogenous GFP-CD63 (*green*) and SiR-Lysosome (*magenta*). (*See also Fig 6G and H*)
