# Supplementary figures and images for: SKIP‐HOPS recruits TBC1D15 for a Rab7‐to‐Arl8b identity switch to control late endosome transport
Source: EMBO J. 2020 Feb 21;39(6):e102301. doi: 10.15252/embj.2019102301 (PMC7073467; doi:10.15252/embj.2019102301)

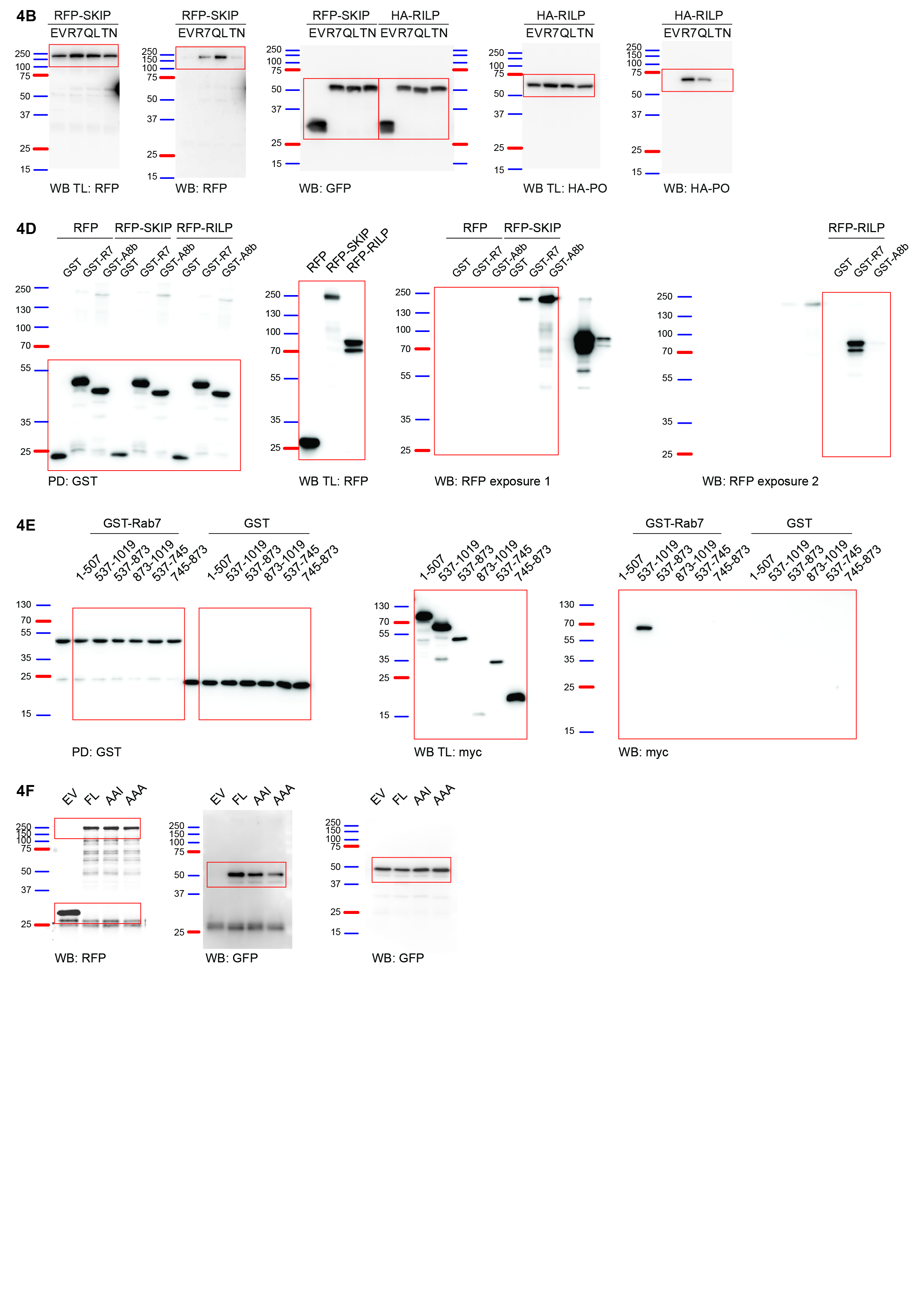

Supplement: Supplementary file 14 — Source Data for Figure 4 [file EMBJ-39-e102301-s012.tif]

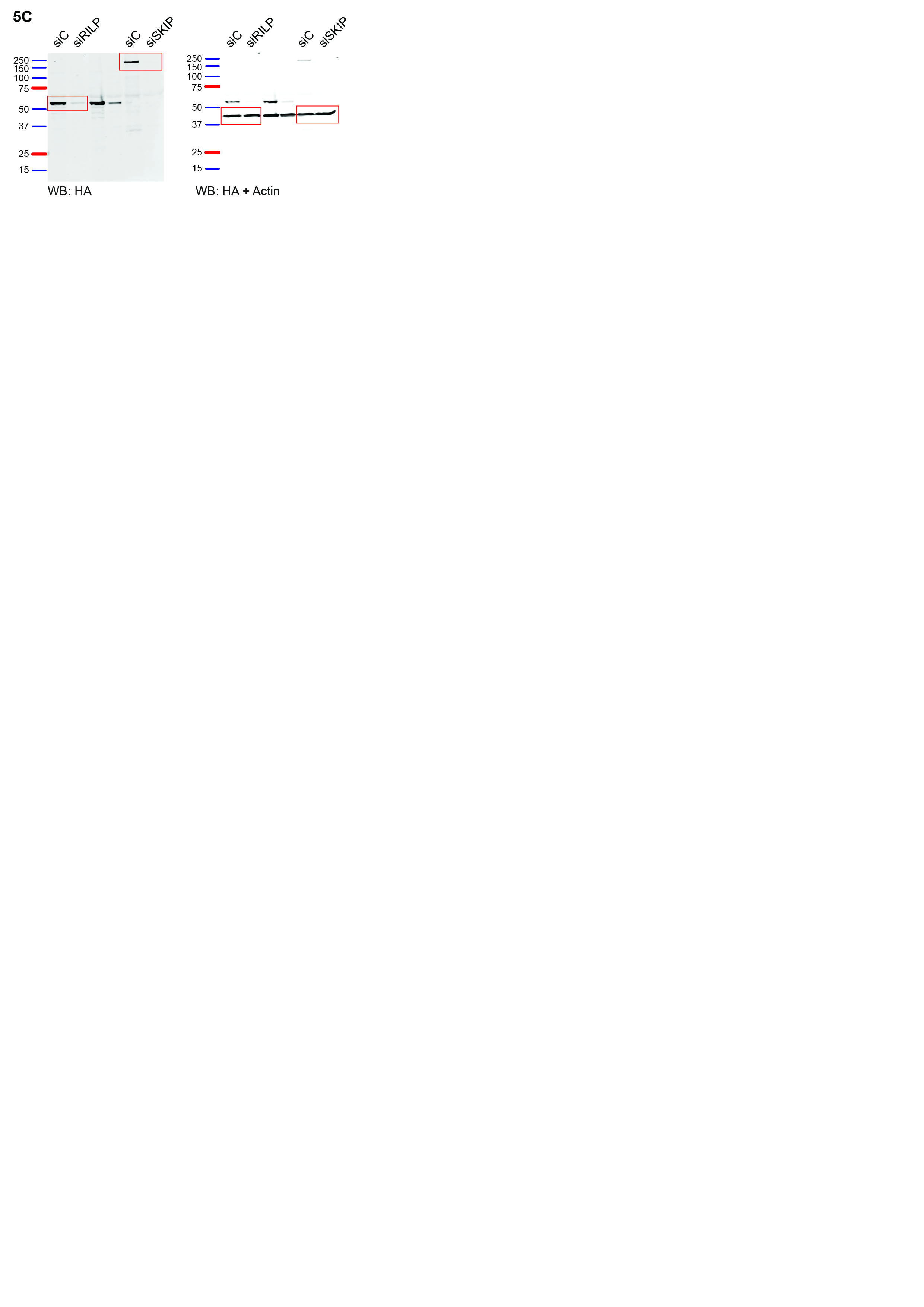

Supplement: Supplementary file 15 — Source Data for Figure 5 [file EMBJ-39-e102301-s013.tif]

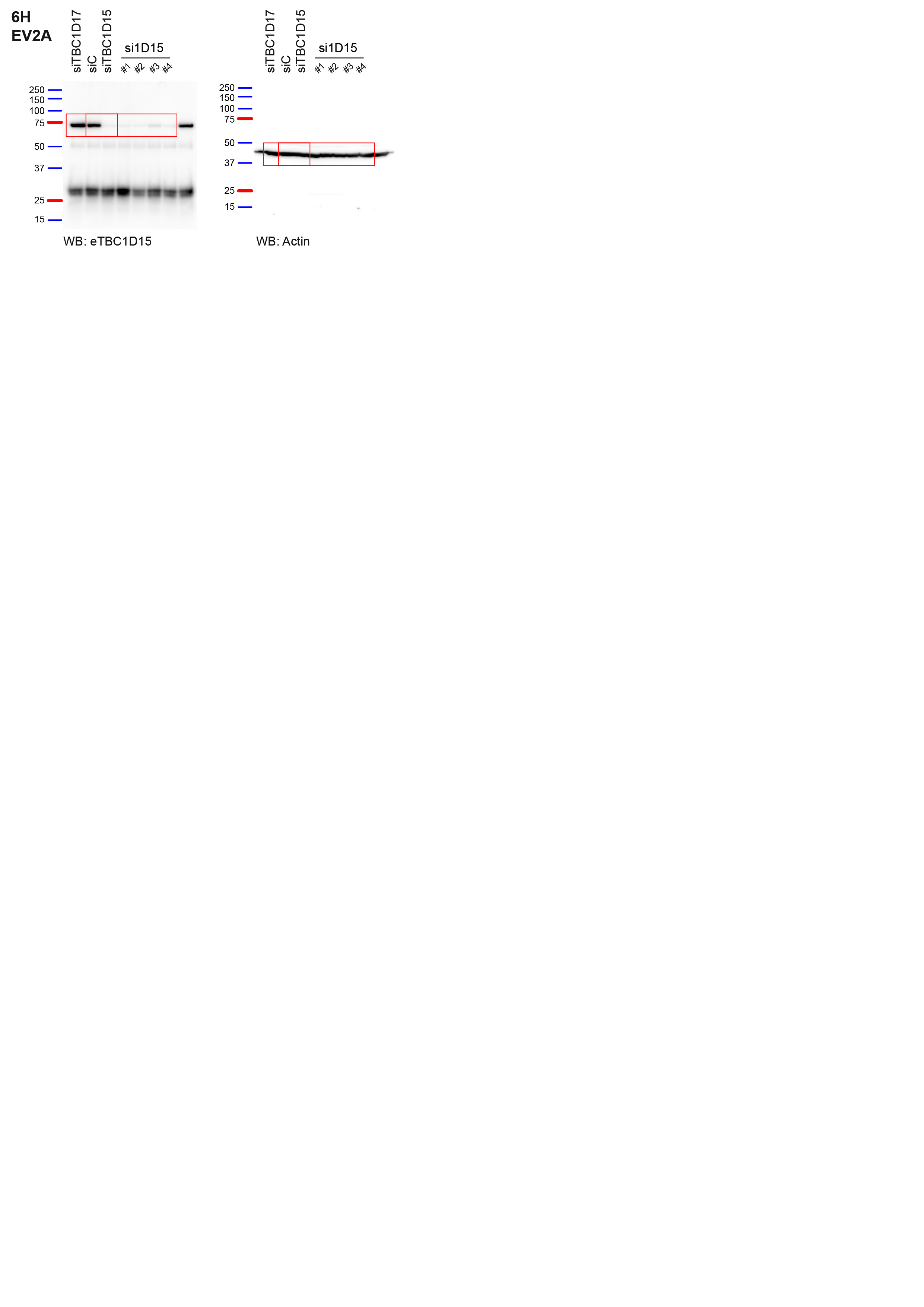

Supplement: Supplementary file 16 — Source Data for Figure 6 [file EMBJ-39-e102301-s014.tif]

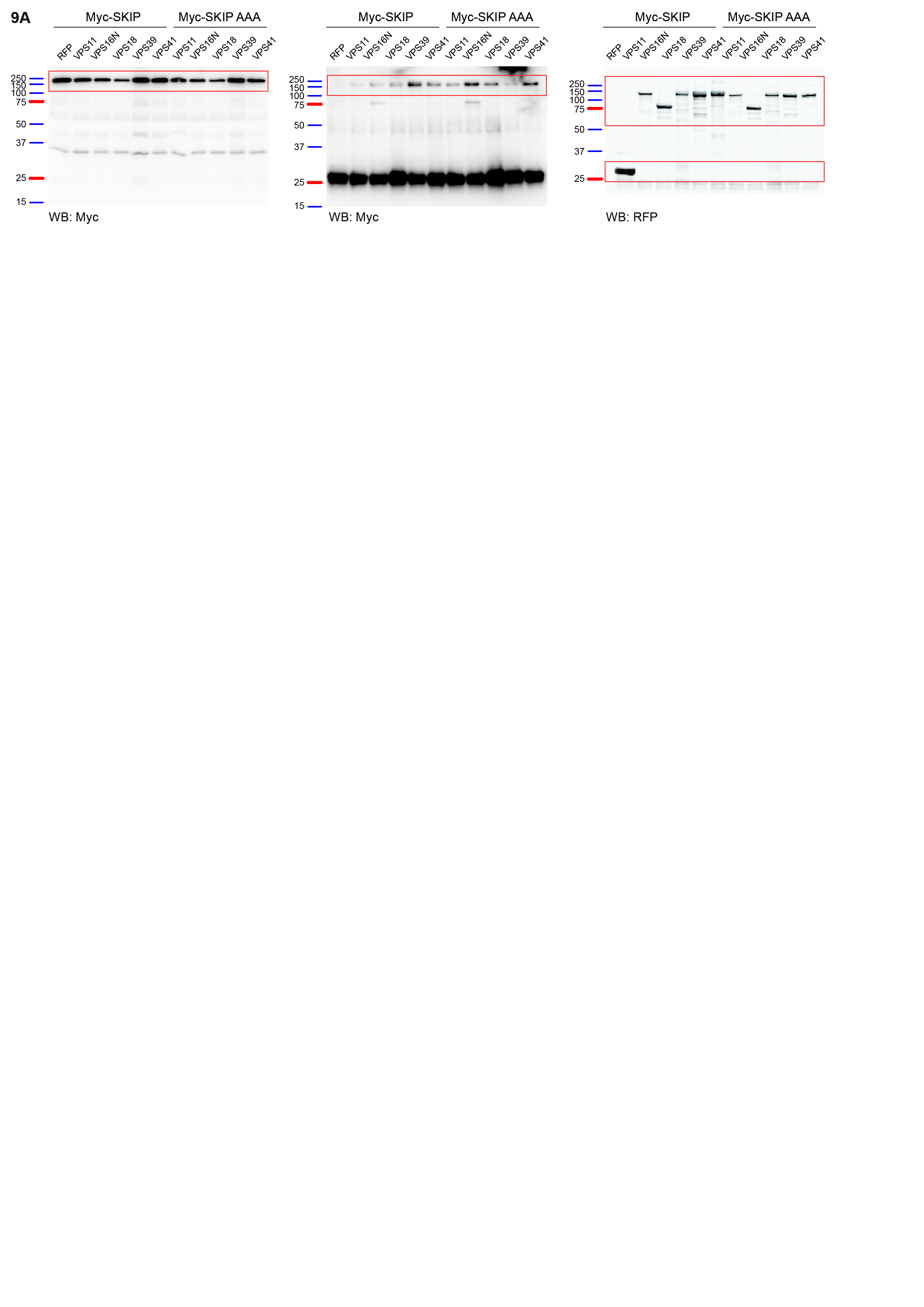

Supplement: Supplementary file 17 — Source Data for Figure 9 [file EMBJ-39-e102301-s015.tif]

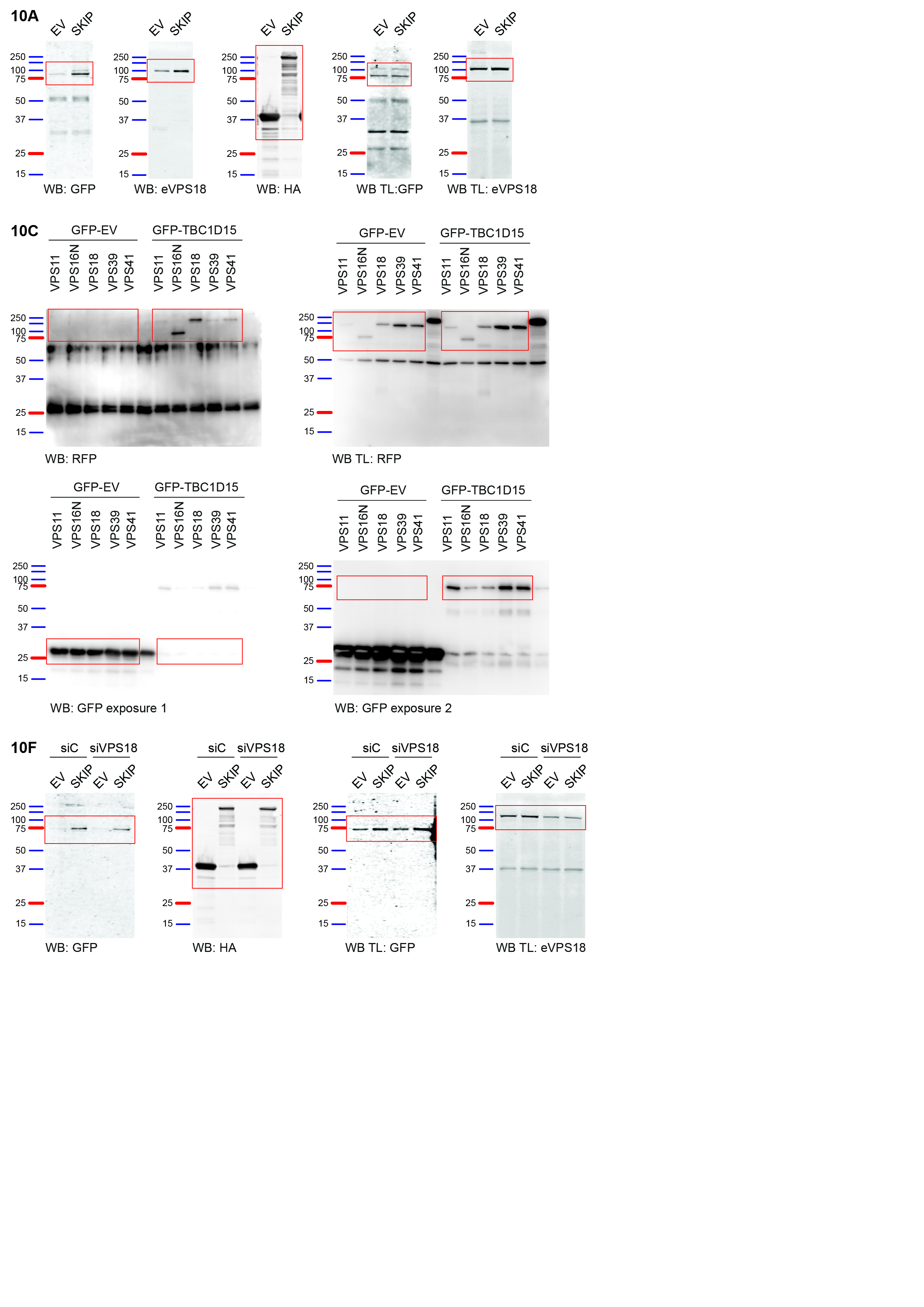

Supplement: Supplementary file 18 — Source Data for Figure 10 [file EMBJ-39-e102301-s016.tif]
