# Supplementary material for: SKIP‐HOPS recruits TBC1D15 for a Rab7‐to‐Arl8b identity switch to control late endosome transport
Source: EMBO J. 2020 Feb 21;39(6):e102301. doi: 10.15252/embj.2019102301 (PMC7073467; doi:10.15252/embj.2019102301)
Supplement: Supplementary file 8 — Movie EV7 [file EMBJ-39-e102301-s008.zip › Movie_EV7B.zip!/Movie Legend EV7B.docx]

**Movie EV7B.** **Recruitment of SKIP to Rab7-positive compartments: example 1 (*related to Figure 4*).**

Single channel view of Movie EV7A. Time-lapse (133 min, 20 s / frame) of HeLa cells co-expressing GFP-ER-SKIP and mCherry-Rab7 (*white*) together with HA-RILP expressed at low levels (cells transfected at 1:5 RILP:SKIP ratio) following Tamoxifen addition.
